# Robotic-assisted versus conventional laparoscopic ICG-fluorescence lymphatic-sparing palomo varicocelectomy: a comparative retrospective study of techniques and outcomes

**DOI:** 10.1007/s00345-024-04909-2

**Published:** 2024-04-06

**Authors:** Ciro Esposito, Ernesto Leva, Marco Castagnetti, Mariapina Cerulo, Mery Cardarelli, Fulvia Del Conte, Giovanni Esposito, Annalisa Chiodi, Marco Chiarenza, Claudia Di Mento, Maria Escolino

**Affiliations:** 1 Pediatric Surgery Unit, Federico II University Hospital, Via Pansini 5, 80131 Naples, Italy; 2 Pediatric Surgery Unit, Ospedale Maggiore Policlinico, Milan, Italy; 3 Pediatric Urology Unit, Bambin Gesù Children Hospital, Rome, Italy; 4 Advanced Biotechnology Center CEINGE, Naples, Italy; 5 Urology Unit, Medical University of Padua, Padua, Italy

**Keywords:** Varicocele, Children, Robotics, ICG, Laparoscopy, Lymphatics

## Abstract

**Purpose:**

This study aimed to compare techniques and outcomes of robotic-assisted varicocelectomy (RAV) and laparoscopic varicocelectomy (LV).

**Methods:**

The medical records of 40 patients, who received RAV and LV over a 2-year period, were retrospectively analyzed. Palomo lymphatic-sparing varicocelectomy using ICG fluorescence was adopted in all cases. Three 5-mm trocars were placed in LV, whereas four ports, three 8-mm and one 5-mm, were placed in RAV. The spermatic vessels were ligated using clips in LV and ligatures in RAV. The two groups were compared regarding patient baseline and operative outcomes.

**Results:**

All patients, with median age of 14 years (range 11–17), had left grade 3 varicocele according to Dubin–Amelar. All were symptomatic and 33/40 (82.5%) presented left testicular hypotrophy. All procedures were completed without conversion. The average operative time was significantly shorter in LV [20 min (range 11–30)] than in RAV [34.5 min (range 30–46)] (*p* = 0.001). No significant differences regarding analgesic requirement and hospitalization were observed (*p* = 0.55). At long-term follow-up (30 months), no complications occurred in both groups. The cosmetic outcome was significantly better in LV than RAV at 6-month and 12-month evaluations (*p* = 0.001). The total cost was significantly lower in LV (1.587,07 €) compared to RAV (5.650,31 €) (*p* = 0.001).

**Conclusion:**

RAV can be safely and effectively performed in pediatric patients, with the same excellent outcomes as conventional laparoscopic procedure. Laparoscopy has the advantages of faster surgery, smaller instruments, better cosmesis and lower cost than robotics. To date, laparoscopy remains preferable to robotics to treat pediatric varicocele.

**Supplementary Information:**

The online version contains supplementary material available at 10.1007/s00345-024-04909-2.

## Introduction

The incidence of varicocele in the healthy male population ranges between 8 and 16% [1]. It is one the most common surgical diseases identified in young men being evaluated for infertility [2]. The negative impact of varicocele on testicular function occurs mainly due to increased oxidative stress within the testicular parenchyma which is thought to be caused by scrotal hyperthermia, testicular hypoxia, and blood–testis barrier disruption [3, 4].

The reduction in testicular temperature after varicocele ligation can improve the fertility rate of these patients. Several studies published in the international literature have reported the significant benefits of surgical treatment of varicocele on semen parameters [5, 6].

Several surgical techniques are available to treat varicocele, using open surgery via inguinal or sub-inguinal approach or using minimally invasive surgery via retro-peritoneoscopic high ligation or laparoscopic ligation [7–11]. The main issue of laparoscopic approach is the high incidence of postoperative hydrocele due to lymphatics injury, reported in up to 20% of operated patients [12, 13]. Several studies recently demonstrated that laparoscopic lymphatic-sparing procedure using ICG can eliminate the risk of postoperative hydrocele [14–17]. While risks and benefits of various techniques have been described, the gold standard for varicocele repair in adolescents has not been clearly defined [18].

A recent systematic review and meta-analysis reported that the laparoscopic lymphatic-sparing technique is characterized by the lowest recurrence rate, incidence of hydrocele and other complications, and no reports of testicular atrophy [19]. In the recent years, robotic-assisted surgery is gaining popularity in the pediatric urology field [20]. Recently, the robotic approach has been described for adolescent varicocelectomy [21–23]. Robotics offers several advantages compared with laparoscopy, including faster learning curve, 3D-magnified vision, and better ergonomics to the surgeon [20].

Analyzing the international literature, no comparative studies between laparoscopic and robotic-assisted varicocelectomy (RAV) using ICG lymphatic-sparing technique are currently available.

This study aimed to compare techniques and outcomes of RAV and laparoscopic varicocelectomy (LV) in the pediatric population.

## Materials and methods

The medical records of 40 patients undergoing surgical treatment of varicocele over the period June 2021 to June 2023 were retrospectively analyzed.

Patients were grouped according to the operative technique: 20 patients receiving RAV were enrolled in G1 and 20 patients undergoing LV in G2.

The surgical procedures were carried out by four independent surgeons, comprising two senior surgeons and two resident surgeons. The senior surgeons had extensive surgical experience, with over 500 laparoscopic procedures and nearly 50 robotic procedures performed annually, respectively. In contrast, the resident surgeons conducted approximately 100 laparoscopic procedures and fewer than 15 robotic procedures per year. Pre-operative work-up included clinical examination and Doppler scrotal ultrasound (US) to assess the degree of varicocele and the testicular volume in all patients.

Patient characteristics evaluated included age and weight, pre-operative degree of varicocele, symptoms, and testicular hypotrophy. Surgical parameters included operative time, intra- and postoperative complications, analgesic requirement, length of stay (LOS), postoperative hydrocele, varicocele recurrence, testicular catch-up growth, cosmetic outcome, and total costs. Operative time was calculated from positioning of sterile drapes till to closure of skin incisions. Postoperative complications were graded according to Clavien–Dindo classification [24]. Cosmetic outcome was scored at 6-month and 12-month follow-ups by two independent evaluating surgeons using a 5-point Likert-type scale (1 = worst; 2 = bad; 3 = fair; 4 = good; 5 = excellent). Follow-up included clinical examination at 1, 3, 6, 12 months and Doppler scrotal US at 12 months postoperatively.

The study received appropriate Institute Review Board (IRB) approval.

### Operative technique

Surgical procedures were performed under general anesthesia with orotracheal intubation. Palomo high ligation of the spermatic bundle using the lymphatic-sparing technique with ICG fluorescence was performed either laparoscopically or robotically. Regarding ICG near-infrared fluorescence (NIRF), the IMAGE1 S™ Rubina® system, manufactured by KARL STORZ SE & Co. KG, Tuttlingen, Germany, was adopted to visualize ICG-NIRF images in LV. Firefly®, from Novadaq imaging system, the software integrated in the DA VINCI Xi Robot, was used for ICG-NIRF visualization in RAV. Regarding ports’ number, three 5-mm trocars were placed in LV. Conversely, four ports, including three 8-mm robotic and one 5-mm assistant laparoscopic, were placed in RAV.

The steps of procedure were the same in both approaches. The only difference was that the vessel ligation was performed using titanium metallic clips in LV and ligatures in RAV.

Video reproduces all steps of the robotic-assisted Palomo technique.

### Statistical analysis

The two groups were compared regarding patient baseline and operative outcomes. Statistical analysis was carried out using the Statistical Package for Social Sciences (SPSS Inc., Chicago, Illinois, USA), version 13.0. The associations between qualitative variables were measured by the *χ*^2^ test and the quantitative variables were measured with the parametric Student's *t* test. *p* < 0.05 was considered statistically significant.

## Results

Median patient age at surgery was 14 years (range 11–17) and median body weight was 55.7 kg (range 29–85). All patients had left grade 3 varicocele according to Dubin–Amelar classification. All were symptomatic (testicular pain and/or discomfort) and 33/40 (82.5%) presented left testicular hypotrophy, defined as testicular size discrepancy of greater than 20% on scrotal US. No significant differences about patient baseline were found between G1 and G2 (Table 1).

**Table 1:** Patient baseline in G1 and G2

Parameter	RAV (G1) *n* = 20	LV (G2) *n* = 20	*p* value
Median age, years (range)	14.4 (12.5–17)	13.6 (11–16)	0.55
Median body weight, kg (range)	57.5 (33–85)	53.9 (29–78)	0.66
Pre-operative varicocele grade			
Dubin–Amelar grade 1, *n* (%)	0	0	n/a
Dubin–Amelar grade 2, *n* (%)	0	0	n/a
Dubin–Amelar grade 3, *n* (%)	20 (100)	20 (100)	0.33
Pre-operative symptoms, *n* (%)	20 (100)	20 (100)	0.33
Pre-operative left testicular hypotrophy, *n* (%)	18 (90)	15 (75)	0.55

*RAV* robot-assisted varicocelectomy, *LV* laparoscopic varicocelectomy

All procedures were completed laparoscopically or robotically without any conversion or intra-operative complications. The average operative time was significantly shorter in G2 [20 min (range 11–30)] than in G1 [34.5 min (range 30–46)] (*p* = 0.001). No technical difficulties to identify and isolating vessels and spare lymphatics were found in both approaches. No adverse local or systemic reactions to ICG were observed.

**Fig. 1:**
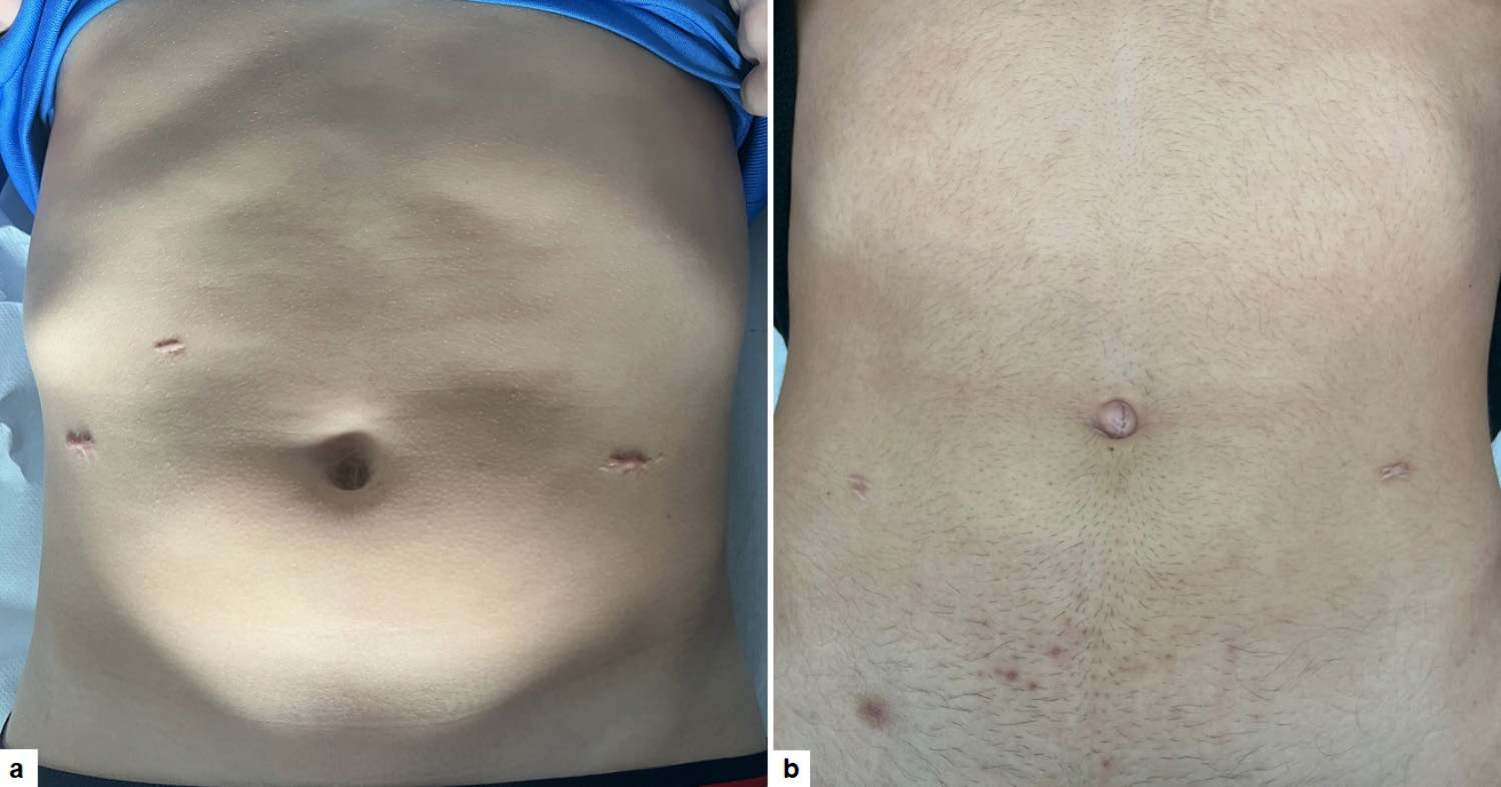
Cosmetic outcome at 1-year follow-up in RAV (**a**) and LV (**b**)

The median analgesic requirement was 14.2 h (range 8–18), without significant differences between the two groups (*p* = 0.55). The median LOS was 20.6 h (range 10–26), without significant differences between the two groups (*p* = 0.55). All patients of both groups were able to resume daily activities on the same day of surgery and full activities within 2 postoperative weeks. At long-term follow-up (30 months), no complications or recurrence of varicocele occurred in both groups. No hematomas, infections, or hydroceles were noted. All patients reported resolution of pre-operative symptoms. Catch-up growth of the affected testicles was noted upon clinical examination and scrotal US in 31/33 (94%) boys who underwent surgery due to reduced testicular size, without significant differences between the two techniques (*p* = 0.33). The cosmetic outcome was significantly better in G2 than G1 at both 6-month and 12-month evaluations (*p* = 0.001) (Fig. 1). The total cost of the laparoscopic procedure was 1.587,07 euros (€) vs. 5.650,31 euros (€) of the robotic-assisted procedure (*p* = 0.001).

Operative outcomes in G1 and G2 are summarized in Table 2.

**Table 2:** Operative outcomes in G1 and G2

Parameter	RAV (G1) *n* = 20	LV (G2) *n* = 20	*p* value
Median operative time, min (range)	34.5 (30–46)	20 (11–30)	**0.001**
Conversion, *n* (%)	0	0	n/a
Intra-operative complications, *n* (%)	0	0	n/a
Visualization of lymphatics on ICG-NIRF, *n* (%)	20 (100)	20 (100)	0.33
Adverse local or systemic reactions to ICG, *n* (%)	0	0	n/a
Median analgesic requirement, hours (range)	13.6 (8–17)	14.8 (10–18)	0.55
Median length of stay, hours (range)	21.5 (12–26)	19.7 (10–22)	0.55
Postoperative complications, *n* (%)	0	0	n/a
Varicocele recurrence, *n* (%)	0	0	n/a
Postoperative hydrocele, *n* (%)	0	0	n/a
6-month cosmetic score, *n* (range)	1.9 (1–3)	3.8 (2–4)	**0.001**
12-month cosmetic score, *n* (range)	3.1 (1–4)	4.8 (3–5)	**0.001**
Testicular catch-up growth, *n* (%)	17/18 (94.4)	14/15 (93.3)	0.33
Total cost of the procedure, euros (€)	1.587,07	5.650,31	**0.001**

*RAV* robot-assisted varicocelectomy, *LV* laparoscopic varicocelectomy

The bold has been used to highlight statistical significance

## Discussion

Analyzing the international literature, it seems that the lymphatic-sparing Palomo procedure is the most adopted technique for treatment of adolescent varicocele [18]. Kass and Marcol [25] demonstrated that the Palomo technique resulted in a significant decrease in the operative failure rate compared to the artery sparing procedures and it should be the preferred technique for varicocele ligation in the adolescent.

As artery sparing varicocelectomy offered no advantage about catch-up growth and was associated with higher incidence of varicocele recurrence, preservation of the artery does not appear to be routinely necessary in adolescent varicocelectomy [26]. Other authors also suggested that the number of arteries identified and preserved with meticulous spermatic cord dissection does not correlate with improvement in semen parameters [27]. Conversely, LV with internal spermatic artery ligation can reduce the recurrence rate and results in the same catch-up growth rate in comparison with LV with spermatic artery preservation [28].

At beginning of experience, the main issue with laparoscopic Palomo procedure was the high incidence of postoperative hydrocele, due to non-selective ligation of the entire spermatic bundle, reported in up to 20% of operated patients [12, 13]. Anatomical studies showed that the mean number of lymphatics in the spermatic cord was around three on both sides [29]. Thus, to avoid risk of postoperative hydrocele, it is necessary to spare at least 1 or 2 lymphatics. Since then, lymphatic-sparing procedures have been adopted to decrease the risk of postoperative hydrocele. Different vital dyes and techniques have been described [17]. The most used dyes were isosulfan blue, first introduced by Chiarenza et al. [30] and indocyanine green (ICG), first described by Esposito et al. [14]. The method of administration of such vital dyes was through intratesticular injection.

In the recent years, robotic-assisted surgery using Da Vinci Xi robotic system has become an alternative to laparoscopy to perform lymphatic-sparing Palomo procedure using ICG-NIRF technology [21–23].

To the best of our knowledge, this is the first comparative study between robot-assisted varicocelectomy and LV using ICG lymphatic-sparing technique.

This analysis showed that postoperative course and outcomes were similar in both groups. The postoperative pain and analgesic requirement were minimal and not significantly different between the 2 procedures. Conversely, the cosmetic outcome was significantly better for laparoscopic procedure rather than robotic-assisted one. The scars of robotic ports were bigger compared to laparoscopic scars (8-mm vs. 5-mm). Also, the number of scars was different between the two techniques (3 in LV vs. 4 in RAV).

Regarding the length of surgery, it was significantly longer in RAV than in LV. However, we must consider that the total operative time of robotic-assisted procedure also included docking time, which ranged between 10 and 20 min, according to the operating team’s experience.

The view of surgical field is much better in robotics, which provides 3-D vision and so as the ergonomics of the console surgeon, who is seated comfortably at robotic console. The ergonomics is not so equally better for the bedside surgeon, who fights with the robotic arms which cover completely the patient leaving a very small space of movement. Robotic platform is also an excellent training tool for young residents and trainees. The double console system allows the main surgeon to be seated at master control while the trainee is seated at the second console and performs the procedure under direct supervision. The findings of the study indicate that robotic surgery facilitated the transfer of surgical skills in minimally invasive techniques even to colleagues with limited laparoscopic and robotic experience.

Both laparoscopy and robotics allow use ICG-NIRF technology to perform real-time lymphography and effective lymphatic-sparing. Following the intra-parenchymal injection of 1 mL ICG solution, the lymphatics become fluorescent green in a matter of 30–60 s. However, after 10 min, we observed diffusion of ICG in spermatic veins and artery, making more difficult visualization of lymphatics. For this reason, the lymphatics dissection and sparing should be completed within 10 min. Lymphatics sparing usually required not more than 2–3 min in our experience. It is worth to perform adequate sparing of lymphatic vessel during ligation according to Palomo to improve surgical outcomes and mitigate the risk of postoperative hydrocele. In our experience, lymphatics sparing was achieved in all patients from both groups and no postoperative hydrocele was observed.

The main drawback of robotics remains the high cost. In our country, the total cost of the laparoscopic procedure was 1.587,07 euros (€) vs. 5.650,31 euros (€) of the robotic-assisted procedure (*p* = 0.001).

The main limitation of this study is the retrospective design. Furthermore, semen parameters were not assessed as most of our patients were younger than 17 years. Further prospective series, including investigation of semen parameters on an individual basis, may help serve as a further incentive for surgery in cases of reduced semen quality.

In conclusion, RAV can be safely and effectively performed in pediatric patients, with the same excellent outcomes as conventional laparoscopic procedure. Robotics provides additional technical benefits such hand tremor filtering, 3D-vision, and increased ergonomics. Laparoscopy has the advantages of faster surgery, smaller instruments, better cosmesis, and lower cost than robotics. To date, laparoscopy remains preferable to robotics to treat pediatric varicocele. Probably soon, along with the miniaturization of robotic instruments and the decrease of associated costs, RAV may become the technique of choice to treat pediatric varicocele.

## Ethical approval

Ethical approval was waived by the local Ethics Committee of Federico II University of Naples, Italy in view of the retrospective nature of the study and all the procedures being performed were part of the routine care.

## Financial interests

The authors have no relevant financial or non-financial interests to disclose.

## Informed consent

Written informed consent was obtained from the parents or legal guardians.

## Supplementary Information

Below is the link to the electronic supplementary material.

Supplementary file1 (MP4 214856 KB)

## Data Availability

The data that support the findings of this study are available from the corresponding author, ME, upon reasonable request.
